# Transcriptional profiling reveals altered biological characteristics of chorionic stem cells from women with gestational diabetes

**DOI:** 10.1186/s13287-020-01828-y

**Published:** 2020-07-25

**Authors:** Liyun Chen, Chung-Teng Wang, Nicholas R. Forsyth, Pensee Wu

**Affiliations:** 1 Guy Hilton Research Centre, School of Pharmacy and Bioengineering, Keele University, Thornburrow Drive, Stoke-on-Trent, UK; 2 Department of Radiation Oncology, Washington University School of Medicine, St Louis, MO USA; 3 Institute of Basic Medical Sciences, College of Medicine, National Cheng Kung University, Tainan, Taiwan; 4 School of Life Science, Guangzhou University, Guangzhou, 510006 China; 5 Academic Unit of Obstetrics and Gynaecology, University Hospital of North Midlands, Stoke-on-Trent, UK; 6 Keele Cardiovascular Research Group, School of Primary, Community, and Social Care, Keele University, Stoke-on-Trent, UK

**Keywords:** Aldehyde dehydrogenase, Chorionic stem cells, Gestational diabetes, Microarray analysis, Migration

## Abstract

**Background:**

Gestational diabetes (GDM) is a common complication of pregnancy. The impact of pregnancy complications on placental function suggests that extraembryonic stem cells in the placenta may also be affected during pregnancy. Neonatal tissue-derived stem cells, with the advantages of their differentiation capacity and non-invasive isolation processes, have been proposed as a promising therapeutic avenue for GDM management through potential cell therapy approaches. However, the influence of GDM on autologous stem cells remains unclear. Thus, studies that provide comprehensive understanding of stem cells isolated from women with GDM are essential to guide future clinical applications.

**Methods:**

Human chorionic membrane-derived stem cells (CMSCs) were isolated from placentas of healthy and GDM pregnancies. Transcriptional profiling was performed by DNA microarray, and differentially regulated genes between GDM- and Healthy-CMSCs were used to analyse molecular functions, differentiation, and pathway enrichment. Altered genes and biological functions were validated via real-time PCR and in vitro assays.

**Results:**

GDM-CMSCs displayed, vs. Healthy-CMSCs, 162 upregulated genes associated with increased migration ability, epithelial development, and growth factor-associated signal transduction while the 269 downregulated genes were strongly linked to angiogenesis and cellular metabolic processes. Notably, significantly reduced expression of detoxification enzymes belonging to the aldehyde dehydrogenase gene families (ALDH1A1/1A2, ALDH2, ALDH3) accounted for downregulation across several metabolic pathways. ALDH activity and inhibitor assays indicated that reduced gene expression of ALDHs affected ALDH enzymatic functions and resulted in oxidative stress dysregulation in GDM-CMSCs.

**Conclusion:**

Our combined transcriptional analysis and in vitro functional characterisation have provided novel insights into fundamental biological differences in GDM- and Healthy-CMSCs. Enhanced mobility of GDM-CMSCs may promote MSC migration toward injured sites; however, impaired cellular metabolic activity may negatively affect any perceived benefit.

## Background

Mesenchymal stem cells (MSCs) are present in many adult tissues and play a role in tissue regeneration and maintenance. Their regenerative potential provides numerous benefits for disease treatment [1]. In terms of differentiation potential toward multiple lineages and their inherent immunomodulatory capacity, MSC from different tissues may share an element of a common transcriptional signature [2]. However, transcriptional profiles have also been shown to be unique for MSCs derived from different tissues [3–5]. Stem cell therapies remain hampered by our incomplete knowledge of these fundamental differences [6]. Unlike embryonic stem cells, the MSC niche in specific adult tissues may affect and determine the gene expression in those specific tissue-derived MSCs [7]. It is highly likely that many genes critical to regenerative functions of MSCs or altered gene signatures associated with different MSC sources are not understood. An improved knowledge of MSC gene profiles would facilitate effective strategies for the use of MSCs in regeneration medicine.

The placenta has a pivotal role in embryogenesis and contains plentiful undifferentiated stem cells and as part of the extraembryonic tissue, and stem cells can be isolated from the placenta without additional invasive procedures or additional ethical concerns [8]. During embryogenesis, the extraembryonic mesoderm surrounding the amniotic cavity gives rise to the placental membrane, composed of the amniotic and chorionic mesoderm [9]. Chorionic MSCs (CMSCs) are derived from the chorion and share common MSC characteristics and multipotency [10, 11]. As CMSCs emerge during pregnancy, their characteristics are highly likely to be affected by pregnancy complications. Gestational diabetes mellitus (GDM) is a common pregnancy complication defined by the new onset of glucose intolerance during pregnancy [12]. The hyperglycaemic environment leads to long-term adverse effects on the offspring and the mother, including increased risk of GDM in subsequent pregnancies and type 2 diabetes in later life [13]. Previous studies have shown that maternal GDM had an adverse effect on the proliferation and viability of umbilical cord-derived MSCs [14], as well as low yield rate of perivascular stem cells from umbilical cords [15]. Chorionic villus-derived mesenchymal stromal cells exhibited decreased clonogenicity and angiogenic potential in GDM compared with healthy pregnancies [16]. However, the underlying molecular events behind those observed differences remain unclear.

A microarray study on umbilical vein endothelial cells derived from GDM pregnancies indicated altered gene expression in insulin sensing and extracellular matrix reorganisation [17]. Gene profiles of umbilical cord tissue from diabetic pregnancies showed alterations in genes associated with vascular development and function [18]. It is noticeable that very few studies have investigated the MSC transcriptional signature altered by pregnancy complications. Not only is gene profiling research on the regenerative ability of MSCs from GDM pregnancies limited, but microarray profiling of CMSCs is yet to be described and made publically available.

With increasing interest in the utilisation of placental MSCs and their banking for clinical purposes, understanding the characteristics and regenerative potential of placental MSCs has become an important subject. Thus, we sought to explore gene expression profiles between CMSCs isolated from healthy and GDM placenta and establish biological differences or similarities. Our findings demonstrate the influence of GDM on CMSC transcriptional profiles with corresponding changes in functionality through in vitro assays. The observed enhanced migration and epithelial development potential in GDM-CMSCs may have clinical benefits for wound healing. On the other hand, the decreased expression and activity of ALDH detoxification enzymes in GDM-CMSCs leads to downregulation of several degradation pathways and an impaired ability to respond to oxidative stress. The comprehensive understanding of GDM-CMSCs reveals the benefits and disadvantages of utilising CMSCs from GDM pregnancies for future regenerative medicine.

## Materials and methods

### Human samples

Placenta samples were obtained with informed written consent and in accordance with procedures approved by the Research Ethics Committee and Health Research Authority (Reference 15/WM/0342). Full-term placentas from 10 healthy and 11 GDM pregnancies were collected from Royal Stoke University Hospital, UK.

### Cell isolation and culture

All placentas were collected immediately after caesarean section, and cell isolation was performed within 1 h. CMSCs were isolated from the chorionic membrane and characterised by immunophenotyping with high levels of expression of typical MSC markers CD73, CD90, and CD105 and low levels of CD14, CD19, CD34, CD45, and HLA-DR. CMSC isolation, as previously described [19], was performed by removing the amniotic membrane and decidual tissue from the chorionic membrane, digesting with 0.05% trypsin/EDTA solution followed by digestion media containing 1 mg/ml collagenase type IV and 25 μg/ml DNase I (Thermo Scientific) in serum-free Dulbecco’s modified Eagle medium (DMEM, Lonza). Cells were cultured in DMEM consisting of 10% foetal bovine, serum 1% l-glutamine, and 1% non-essential amino acids (NEAA, Lonza). The CMSC samples used in DNA microarray were from 3 healthy and 3 GDM pregnancies at passage 3. Other samples were used for validation of microarray data by analysis gene expression through qPCR and functional assays.

### DNA microarray

0.2 μg of total RNA was amplified and labelled with Cy3 (CyDye, Agilent Technologies) for in vitro transcription process. Labelled cRNA was pooled and hybridised to Agilent SurePrint Microarray (Agilent Technologies) according to the manufacturer’s protocol. Arrays were scanned with an Agilent microarray scanner and images analysed by Feature extraction10.7.3.1 software (Agilent Technologies).

### Microarray analysis using bioinformatics software

Venn diagram and hierarchical clustering heat maps were created by AltAnalyze software (Gladstone Institution, UCSF) and used to identify commonly upregulated or downregulated genes and illustrate the differentially expressed gene lists of interest.

Ingenuity Pathway Analysis (IPA, Qiagen; www.qiagen.com/ingenuity) was used to analyse genes with fold changes > 1.5 in GDM samples vs. healthy samples. Overrepresented and underrepresented biological functions and canonical pathways were identified based on the selected genes using Ingenuity Knowledge Database (Qiagen). The *p* value, calculated with the Fisher’s exact test, indicates the likelihood that the association between a set of genes in the experimental dataset and a biological function or pathway is the result of random chance. The *p* value < 0.05 indicates a statistically significant. *z*-score based on the match of target genes and biological pathway/function; expression changes of these target genes and their agreement with literature finding predicted the up/downregulation patterns.

Pathway network visualisation was created by Cytoscape v.3.6.1. Enriched gene sets identified by IPA pathway analysis were selected and used as input nodes. The interaction network was generated according to literature findings and public database. The network was manually curated and distributed with circles for easier visualisation.

### RNA extraction and real-time PCR

Total RNA were isolated at passage 3, using TRIzol Reagent (Invitrogen) according to the manufacturer’s instruction. Reverse transcription was performed with High-Capacity cDNA Reverse Transcription Kit (Thermo Scientific). Gene expression analysis was evaluated by real-time PCR using QuantiFast SYBR Green PCR Kit (Qiagen). Primer sequences are shown in Table 1. The relative expression levels of mRNA were normalised to *GAPDH*, and fold change was calculated using the 2^-(ΔΔCt) method.

**Table 1:** Primer sequences

Genes	Forward sequence	Reverse sequence
CD24	CTCCTACCCACGCAGATTTATTC	AGAGTGAGACCACGAAGAGAC
AQP1	CTGCATGGTCAAGCCTCTTA	TCAAGGGAGTGGGTGAATTG
FLNB	TGATCTATGTGCGCTTCGGT	GACATGCATTTACCGGTGCC
CELSR1	TACTTCTGCGGTGCTGGTTT	GTCCGTAAACCGTCCCTTCC
EDN1	CCATGAGAAACAGCGTCAAATC	CGAAGGTCTGTCACCAATGT
HBEGF	AATCTGGCTTAGTGCCACCC	GCACTCTGACCACGGAAGAT
TGFB2	ATGCGGCCTATTGCTTTAGA	ACCCTTTGGGTTCGTGTATC
CTCF	GCCTGTTCCAAGACCTGTG	GGCGGCTCTGCTTCTCTA
NKX2.5	CAACATGACCCTGAGTCCCC	TAATCGCCGCCACAAACTCT
NOG	CATGCCGAGCGAGATCAAA	CAGCCACATCTGTAACTTCCTC
PDGFA	GGAACGCACCGAGGAAGA	GCCAGGAGGAGGAGAAACAG
NPPB	TGGAAACGTCCGGGTTACAG	GACTTCCAGACACCTGTGGG
MET	TGGTGCAGAGGAGCAATGG	CATTCTGGATGGGTGTTTCCG
CXCL12	ATGAACGCCAAGGTCG	GGGCTACAATCTGAAGGG
RASIP1	CGTCTCCTTGAGAACCAATACC	CATTCCACGCGGGATAAGAA
RSPO3	CACCTTTATCTGAGCCAATGGA	ATGCAGGGGGATCTGACATA
HMOX1	TCTTGGCTGGCTTCCTTACC	GGATGTGCTTTTCGTTGGGG
ALDH1A1	TCAAACCAGCAGAGCAAACT	TAGGCCCATAACCAGGAACA
ALDH2	CTGCTGACCGTGGTTACTT	CTCCCAACAACCTCCTCTATG
ALDH3B1	GCTGAAGCCATCGGAGATTAG	GCTCCCTGTGAAGAAGATGTAG
NQO1	GGGATGAGACACCACTGTATTT	AGTGATGGCCCACAGAAAG
SOD2	GGACAAACCTCAGCCCTAAC	GCCGTCAGCTTCTCCTTAAA
GAPDH	ACTTCAACAGCACACCCACT	GCCAAATTCGTTGTCATACCAG

### Transwell migration assay

Cells were placed onto the upper chamber of a Transwell filter with 8-μm pores (Corning), and the bottom well contained regular growth media culturing in a 37 °C incubator. After 8 and 24 h, migrated cells were fixed with methanol and stained with crystal violet. Migration was quantified by cell counts in five separate fields per sample and expressed as mean numbers. Data represent 6 independent experiments in each group, performed in triplicate.

### Wound healing assay

5 × 10^5^ cells were seeded into each well of a 6-well plate and cultured for 2 days. Wounds were created by scratching with a 200-μl pipette tip, and media were changed to remove suspension cells. After incubating for 12 h, cells were visualised under a light microscope. Relative cell migration was calculated by measuring the final wound area compared to the initial area. Three non-overlapping fields were picked and examined per well. Data represent 6 independent experiments in each group.

### ALDH activity analysis

ALDH function was measured by aldehyde dehydrogenase activity colorimetric assay kit (Sigma). Acetaldehyde is oxidised by ALDH generating NADH which reacts with a probe and the activity of ALDH measured by absorbance reading at 450 nm. Cells were lysed by ALDH buffer and manufacturer’s protocol followed. Absorbance was measured every 5 min over a 30-min period. The activity of ALDH was calculated according to the manufacturer’s suggestion. All samples were performed in triplicate.

### Detection of intracellular ROS

ROS was detected by 2′,7′-dichlorofluorescin diacetate (DCFDA, also known as H_2_DCFDA) cellular reactive oxygen species detection kit (Abcam). Cells were seeded at 10,000 cells/well in a 96-well plate. After attachment, cells were incubated with 25 μM DCFDA for 45 min at 37 °C and then treated with 5 mM or 25 mM glucose. For fluorescent images, ROS detection was observed by a confocal microscope. Images were taken from randomly chosen fields in each experiment. The intensity of fluorescence was measured by a microplate reader, and the signal was read at excitation/emission: 485/535 nm. A blank well containing no cells (media only) was used as a background signal. All samples were performed in triplicate.

### Statistics

All statistics were calculated by GraphPad Prism 6 software. Student’s *t* test was used to compare paired or unpaired data. Values are presented as mean ± SEM, and *p* < 0.05 is determined as significant: *p < 0.05, **p < 0.01, and ***p < 0.001.

## Results

### Identification of differentially expressed genes and biological function analysis

**Fig. 1:**
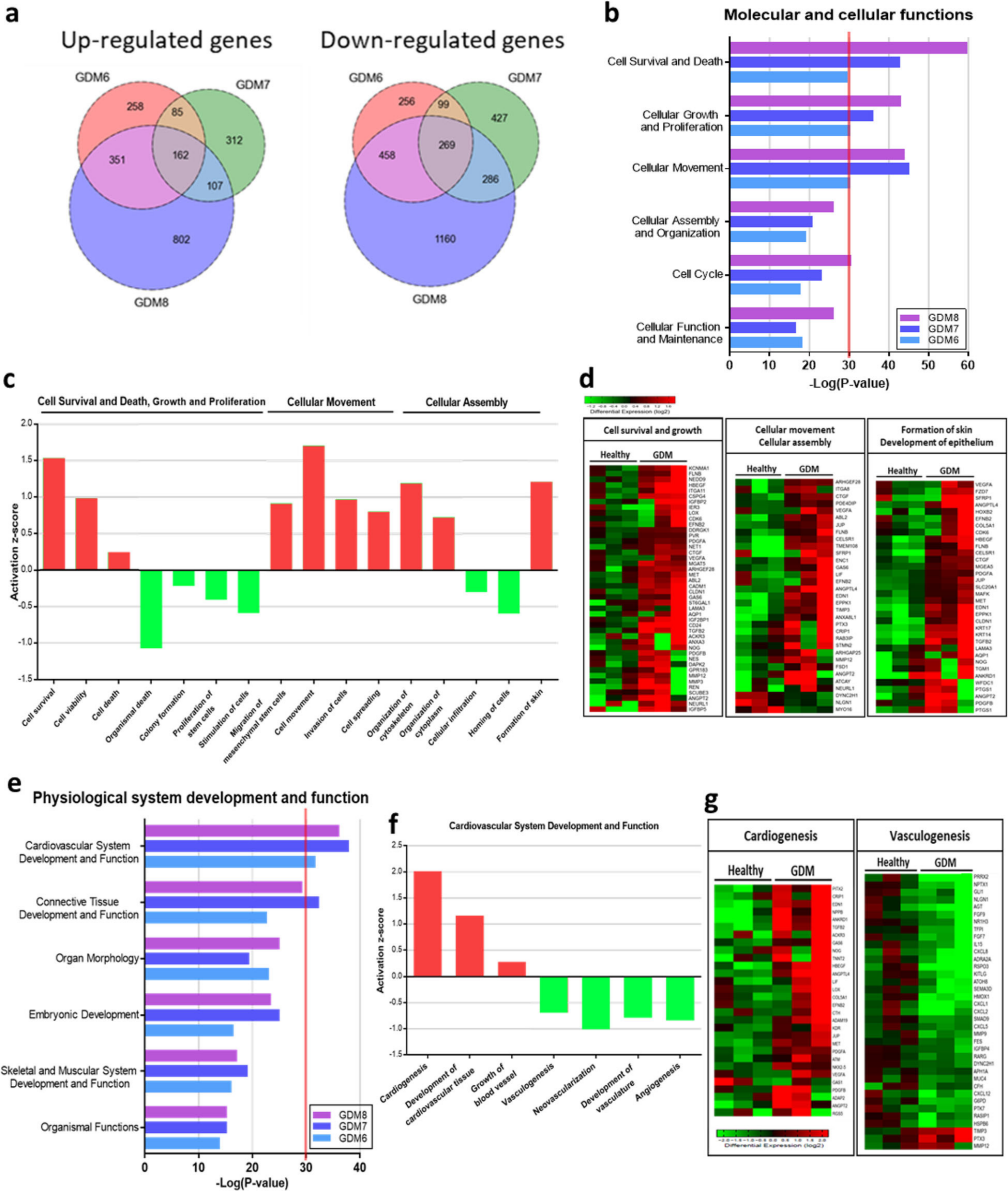
Identification of DEGs and enriched biological functions in GDM-CMSCs. **a** Venn diagram indicates the numbers of genes up- or downregulated > 1.5-fold in GDM-CMSCs vs. Healthy-CMSCs. The overlapping areas of the 3 circles are co-regulated genes and defined as differentially expressed genes (DEGs). **b** Biological functions in “molecular and cellular functions” category were generated and ranked by IPA analysis. Bars indicate overrepresented functions in GDM-CMSCs compared to H-CMSCs. Individual GDM-MSC samples are represented via specific indicated colours. **c** Activation state of enriched downstream cellular processes in “molecular and cellular functions” using IPA activation *z*-score identification of increased (positive *z*-score) or decreased activity (negative *z*-score) in GDM-CMSCs compared to Healthy-CMSCs. **d** Heat maps of DEGs involved in cell survival and growth, cellular movement and assembly, skin formation, and epithelial development. Expression levels are represented by log_2_ fold change (expression value in each sample vs. mean expression value in Healthy-CMSCs). Expression levels range from high (red) to low (green). **e** Biological functions in “physiological system development and function” category in GDM-CMSCs identified by IPA. **f** Cellular processes associated with “cardiovascular system development and function” in GDM-CMSCs. The activation state was calculated by IPA activation *z*-score. **g** Heat maps summarised DEGs involved in cardiogenesis and vasculogenesis

Gene expression profiles of CMSCs derived from 3 healthy (H-CMSCs) and 3 GDM women (GDM-CMSCs) were determined by DNA microarray analysis. Validation of microarray results was performed with 10 H-CMSCs and 11 GDM-CMSCs samples. Applying a cut-off of *p* < 0.05 and 1.5-fold change between H-CMSCs and GDM-CMSCs, we identified a total of 431 differentially expressed genes (DEGs), including 162 upregulated and 269 downregulated genes in all 3 GDM samples (Fig. 1a). The 3 GDM women were treated for their GDM with either metformin (GDM6), insulin (GDM7), or both (GDM8). The sample from the women who received both metformin and insulin treatment exhibited the highest number of total DEGs.

Biological function analysis using DEGs identified in all 3 GDM-CMSCs samples was performed with Ingenuity Pathway Analysis (IPA) focussed on two categories: “molecular and cellular functions” and “physiological system development and functions”. Applying a cut-off of average -log(*p* value) > 30 using Fisher’s exact test, the most represented “molecular and cellular functions” in GDM-CMSCs were related to cell death and survival, cellular growth and proliferation, and cellular movement (Fig. 1b). To further identify the altered downstream cellular process of the most represented biological functions, the activation *z*-score computed by IPA was used to infer the activation/inhibition state and gene enrichment. A positive *z*-score indicates increased functional activity in GDM-CMSCs relative to H-CMSCs while a negative *z*-score indicates a reduction in activity. Positively associated cellular processes in GDM-CMSCs included cell survival, viability, cellular migration and movement, assembly and organisation of cytoskeleton, and skin formation, which are critical functions in wound repair and tissue remodelling (Fig. 1c). DEGs in GDM-CMSCs associated with the positively regulated cellular processes were displayed by gene clustering heat maps, indicating the upregulated expression levels and increased wound healing and remodelling potential (Fig. 1d). Noticeably, organismal death was the most downregulated cellular process in GDM-CMSCs while other negatively associated functions including stimulation of cells, homing, and colony formation showed less significant *z*-score values (Fig. 1c).

### Differentially expressed genes in GDM-CMSCs are significantly involved in cardiovascular system development

In terms of the tissue regenerative potential of GDM-CMSCs, the most represented biological function in the “physiological system development” category was cardiovascular system development and function, which had an average -log(*p* value) > 30 (Fig. 1e). Further in-depth analysis of the downstream functional activation performed in relation to cardiovascular system development showed that cardiogenesis was the most significantly overrepresented downstream cellular process with the highest positive *z*-score, along with the positive association with development of cardiovascular tissue (Fig. 1f). The heat map illustrated a set of genes involved in cardiogenesis that were highly expressed in GDM-CMSCs compared to H-CMSCs, suggesting the greater potential of GDM-CMSCs in cardiac regeneration (Fig. 1g). In contrast, the cellular processes of vasculogenesis and angiogenesis in cardiovascular development had decreased activation with negative *z*-scores (Fig. 1f). The heat map showed the downregulation of vasculogenesis and vasculature development-associated genes in GDM-CMSCs compared with H-CMSCs (Fig. 1g). Moreover, other enriched downstream cellular processes in the “physiological system development” category included respiratory system development and formation of the lung and kidney with a positive *z*-score while development of the exocrine gland and connective tissue had negative *z*-scores in GDM-CMSCs (Additional file 1: Figure S1).

### Validation of increased migration ability, wound healing potential, and cardiac development in GDM-CMSCs

**Fig. 2:**
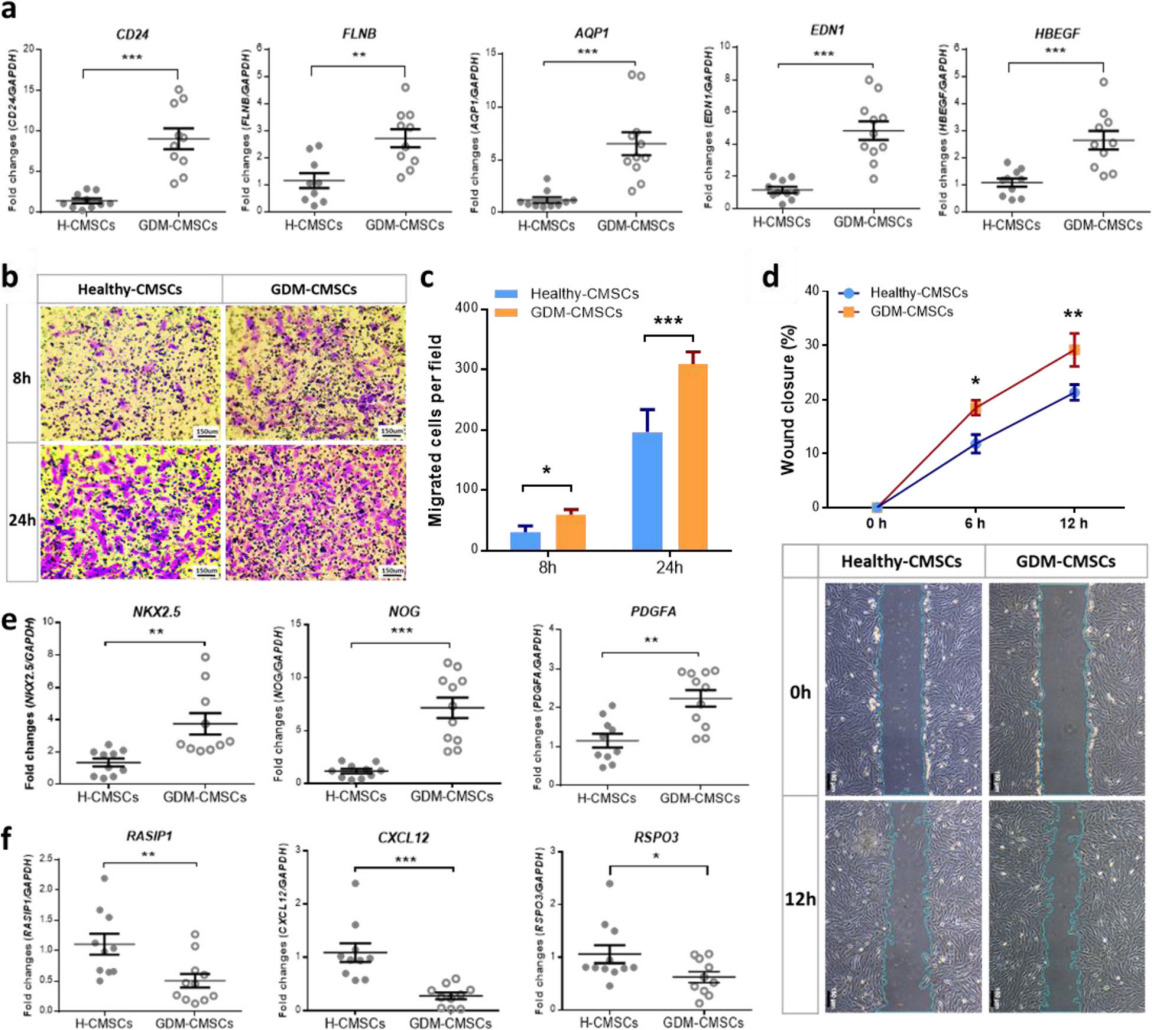
Validation of gene expression and altered biological functions in GDM-CMSCs through real-time PCR and in vitro migration assays. **a** Upregulation in genes associated with cell migration/cellular assembly (*CD24*, *FLNB*, *AQP1*) and wound healing (*EDN1*, *HBEGF*) in GDM-CMSCs validated by real-time PCR with 10 Healthy- and 11 GDM-CMSCs samples. Gene expression levels were normalised to *GAPDH* and presented as fold change by comparing G-CMSCs to H-CMSCs using 2^-(ΔΔCt) method. **b** Motility of cell was evaluated by Transwell migration assay. Representative images of migrated Healthy-CMSCs and GDM-CMSCs stained with crystal violet after 8 and 24 h of migration period. Scale bar 150 μm. **c** Cell migration ability was calculated by counting migrated cells per field by ImageJ. Data represent 6 independent experiments in triplicate. **d** The graph indicates wound closure percentages, and the images below are representative images of wound healing assay. Healthy-CMSCs and GDM-CMSCs migrated into the middle wound field after 12 h. The percentage of wound closure was calculated by measuring the reduced wound area after 6 h and 12 h by ImageJ. Data were obtained from 6 independent experiments. Scale bar 150 μm. **e** Significant upregulation of cardiogenic genes in GDM-CMSCs. Validation of *NKX2.5*, *NOG*, and *PDGFA* expression in healthy and GDM samples was examined by real-time PCR. The expression level of each gene was normalised to *GAPDH*. **f** Significant downregulation of vasculogenic genes in GDM-CMSCs. *RASIP1*, *CXCL12*, and *RSPO3* expressions were validated by real-time PCR. The expression level of each gene was normalised to *GAPDH*. All error bars in this figure are presented as mean ± SEM. Student’s *t* test was used for statistical significance, *p < 0.05, **p < 0.01, ***p < 0.001

To evaluate functional differences between H-CMSCs and GDM-CMSCs, elevated expression of genes involved in migration, survival, and cellular assembly ability in GDM-CMSCs were validated through real-time PCR and migration assays. DEGs associated with cell migration (*CD24*, *AQP1*), cellular assembly (*FLNB*, *CELSR1*), and skin formation and healing process (*EDN1*, *HBEGF*, *TGFB2*, *CTGF*) were significantly upregulated in GDM-CMSCs compared to H-CMSCs (Fig. 2a and Additional file 2: Figure S2a). Transwell migration was performed to examine cell motility, where H-/GDM-CMSCs were placed into the upper compartment of a Transwell filter and allowed to migrate through the filter for 8 and 24 h. After either 8 or 24 h of incubation, enhanced migration ability was observed in GDM-CMSCs with a greater number of cells having migrated across the membrane (Fig. 2b, c). In wound healing assays, higher numbers of GDM-CMSCs migrated into the wound field at every observed time point (6, 12, 24 h) than H-CMSCs. The closure percentages were significantly increased, with approximately 30% in GDM-CMSCs and 20% in H-CMSCs after 12 h (Fig. 2d and Additional file 3: Figure S3). Collectively, both the Transwell migration and wound healing assay validated the enhanced migration ability in GDM-CMSCs.

Cardiovascular development was identified as the most enriched biological function in GDM-CMSCs with altered gene expression in cardiogenesis and vasculogenesis. The expression of genes associated with cardiogenesis (*NKX2.5*, *NOG*, *PDGFA*, *NPPB*, *MET*) showed significant increases in GDM-CMSCs vs. H-CMSCs (Fig. 2e and Additional file 2: Figure S2b). The opposite trend was found in vasculogenesis-associated genes, which showed a significantly reduced expression of *RASIP1*, *CXCL12*, *RSPO3*, and *HMOX1* in GDM-CMSCs (Fig. 2f and Additional file 2: Figure S2b). Taken together, CMSCs derived from GDM placenta may therefore have better potential application in wound repair and cardiogenesis than in vasculogenesis when compared to H-CMSCs.

### Pathways mediating cardiac development and cell movement are both upregulated in GDM-CMSCs

**Fig. 3:**
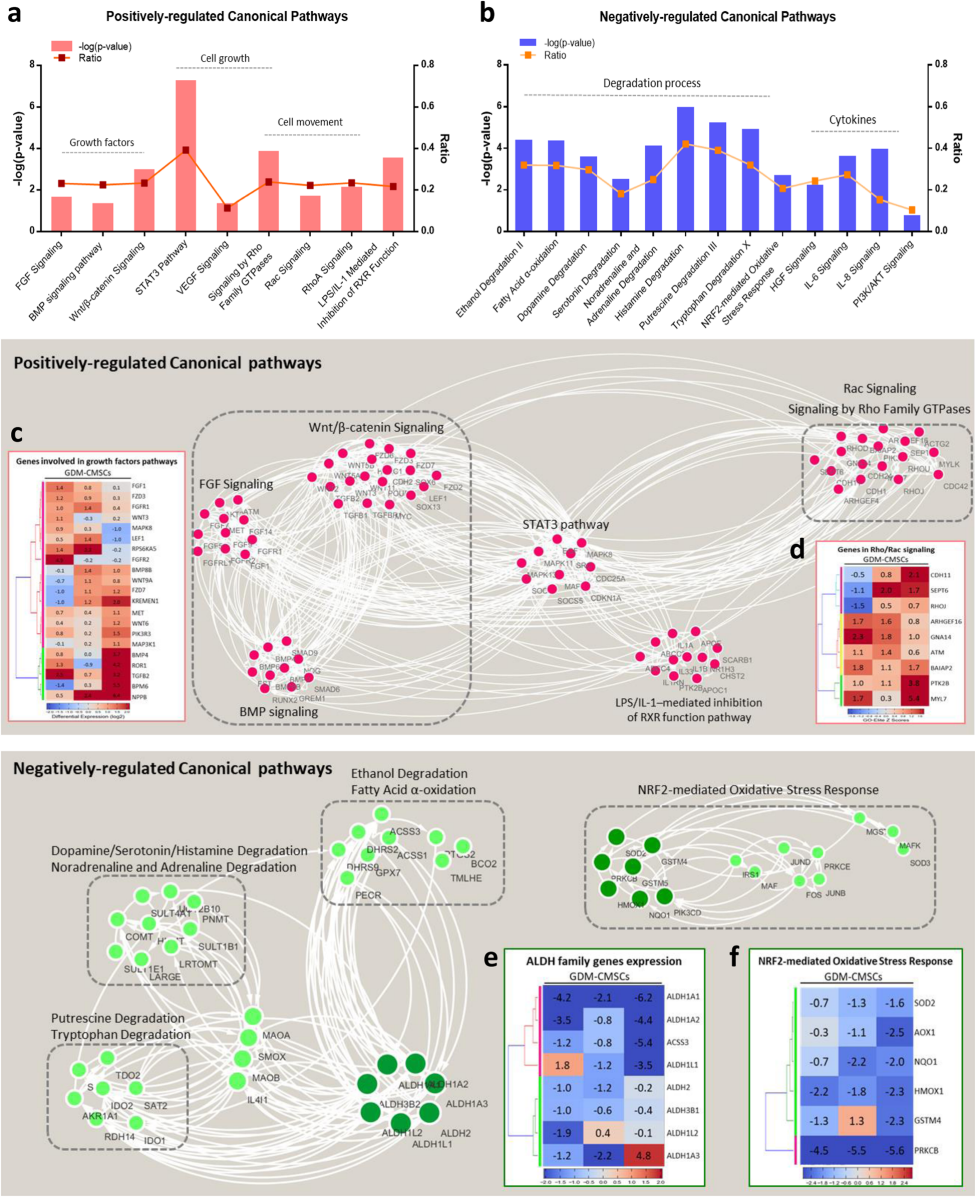
Positively and negatively regulated pathway analysis and regulatory network visualisation. **a**, **b** IPA canonical pathway analysis of positively and negatively regulated pathways in GDM-CMSCs. The *x*-axis indicates the altered canonical pathways in GDM-CMSCs. The left *y*-axis indicates the statistical significance *p* value, calculated using Fisher’s exact test. The right *y*-axis represents the ratio of the number of dataset genes that map to all known pathway genes. **c**, **d** Positively regulated canonical pathways and associated genes. Hierarchical clustering maps indicate the expression of genes involved in BMP, Wnt/β-catenin, FGF signalling, and Rho family signalling. From left to right on the clustering maps are presenting as GDM6, GDM7, and GDM8 samples. Each gene is normalised to a mean expression of 3 Healthy-CMSCs samples. Blue and red indicate below and above mean expression, respectively, with values indicating log_2_ fold change. **e**, **f** Negatively regulated canonical pathways and associated genes. Interaction networks were generated by Cytoscape visualisation based on DEGs. Hierarchical clustering map showed a significant reduction in ALDH family gene expression in GDM-CMSCs and regulators of the Nrf2 pathway

Gene regulatory networks were examined incorporating DEGs for positively regulated (Fig. 3a) and negatively regulated pathways (Fig. 3b). The STAT3 pathway, with roles in development, cellular homeostasis, cell growth, proliferation, and differentiation [20], was the most significantly upregulated signalling pathway in GDM-CMSCs with the highest ratio and -log(*p* value). The activation of pathways such as BMP signalling, Wnt/β-catenin signalling, and FGF signalling which were upregulated in GDM-CMSCs may transduce through STAT3 signalling and contribute to the regulation of various development processes. Multiple upregulated genes in GDM-CMSCs were involved in BMP, Wnt/β-catenin, and FGF signalling (Fig. 3c). Additionally, BMP, Wnt/β-catenin, and FGF signalling play an important role in heart development. As the cardiac-associated genes, *NKX2.5* and *NOG*, are regulated downstream to BMP, Wnt/β-catenin, and FGF pathways, the previously observed upregulation of *NKX2.5* and *NOG* in GDM-CMSCs may contribute to enhanced cardiogenesis potential via the modulation of the BMP, Wnt/β-catenin, and FGF pathways. Other regulatory factors, such as *WNT3/11*, *LEF1*, and *FZD* in the Wnt/β-catenin signalling pathway and *FGF1*, *MAPK*, and *MET* in the FGF signalling pathway, also displayed higher expression in GDM-CMSCs than in H-CMSCs (Fig. 3c).

Moreover, the increased activity of Rho family GTPase signalling in GDM-CMSCs, including Rac, RhoA, and Cdc42 signalling, associates with the regulation of cell migration, invasion, and cytoskeleton organisation [21]. The clustering map showed an increased expression of genes involved in Rho family signalling in GDM-CMSCs, suggesting that enhanced cell movement capacity in GDM-CMSCs may be associated with activation through Rho family signalling (Fig. 3d). Nuclear receptor signalling via LPS/IL-1-mediated inhibition of RXR function pathway also showed an increased activity in GDM-CMSCs.

### Significantly reduced expression in ALDH family genes results in a negative association with metabolic pathways in GDM-CMSCs

Among the downregulated metabolic pathways, most were associated with degradation processes, including ethanol degradation, oxidative ethanol degradation, and fatty acid α-oxidation as well as the degradation of neurotransmitters (histamine, dopamine, noradrenaline, serotonin) and other molecules (putrescine, tryptophan) (Fig. 3b). Given the dysfunctional metabolic regulation in women with GDM, these pathways are of particular interest for investigating GDM-CMSCs behaviours. Figure 3e illustrates the genes associated with these enriched downregulated pathways and showed that the decreased activities of degradation pathways were connected to the significant reduction in aldehyde dehydrogenase family gene expression, *ALDH1*, *ALDH2*, and *ALDH3* (Fig. 3e). Aldehydes can be formed during the metabolism of amino acids, carbohydrates, lipids, and vitamins as well as cytotoxic drugs and environmental chemicals [22]. ALDH genes encode the key enzymes that regulate cellular detoxification through oxidation of endogenous and exogenous aldehydes, and play an important role in many degradation processes [23]. Notably, *ALDH1A1* is one of the most downregulated genes in GDM-CMSCs (Table 2). Other genes including *ALDH1A2*, *ALDH2*, and *ALDH3B1* were also significantly reduced in GDM-CMSCs microarray data and comprised the core molecules of the downregulated pathway network. The deficiency of ALDH family genes may lead to insufficient detoxification resulting in aldehyde accumulation and reactive oxygen species (ROS) imbalance. Additionally, a critical pathway for ROS regulation, the Nrf2-mediated oxidative stress response pathway, showed downregulation in GDM-CMSCs, owing to the low expression of important regulators, protein kinase (*PRKC*), and antioxidant enzyme genes (*HMOX1*, *SOD2*, *AOX1*) (Fig. 3f).

**Table 2:** Top 10 upregulated and downregulated genes in GDM-CMSCs vs. Healthy-CMSCs

Top 10 downregulated genes	Average log_2_ ratio
PRKCB	− 5.566
GJA8	− 4.166
CXCL12	− 3.436
FAM20A	− 3.379
ALDH1A1	− 3.315
PRRX2	− 3.227
ATP8B4	− 3.040
RSPO3	− 2.927
CXCL3	− 2.925
CXCL1	− 2.914
Top 10 upregulated genes	Average log_2_ ratio
RPS4Y1	9.873
COL17A1	9.344
DDX3Y	8.528
CDH1	8.376
KRT5	8.308
SCEL	8.207
SPRR3	7.823
PLD5	7.293
HAPLN1	7.102
L1TD1	6.996

Other downregulated pathways, such as “IL-6 signalling” and “HGF signalling”, regulate multiple molecules involved in angiogenesis (Fig. 3b). For instance, *CEBPB*, *FOS*, *ELK1*, and *CXCL8* in angiogenesis pathways displayed low expression in GDM-CMSCs. Moreover, the downregulation of “PI3K/AKT signalling” may affect the potent angiogenic factors, IL-6, IL-8, HGF, and VEGF signalling transduction through mediation of the PI3K/AKT pathway to promote angiogenesis [24–26]. Biological function analysis identified downregulated genes in angiogenesis and vasculogenesis as downstream components of IL-6, IL-8, and HGF pathways suggesting a regulatory network of reduced angiogenic potential in GDM-CMSCs.

### Reduced ALDH activity in GDM-CMSCs is associated with reactive oxygen species dysregulation

**Fig. 4:**
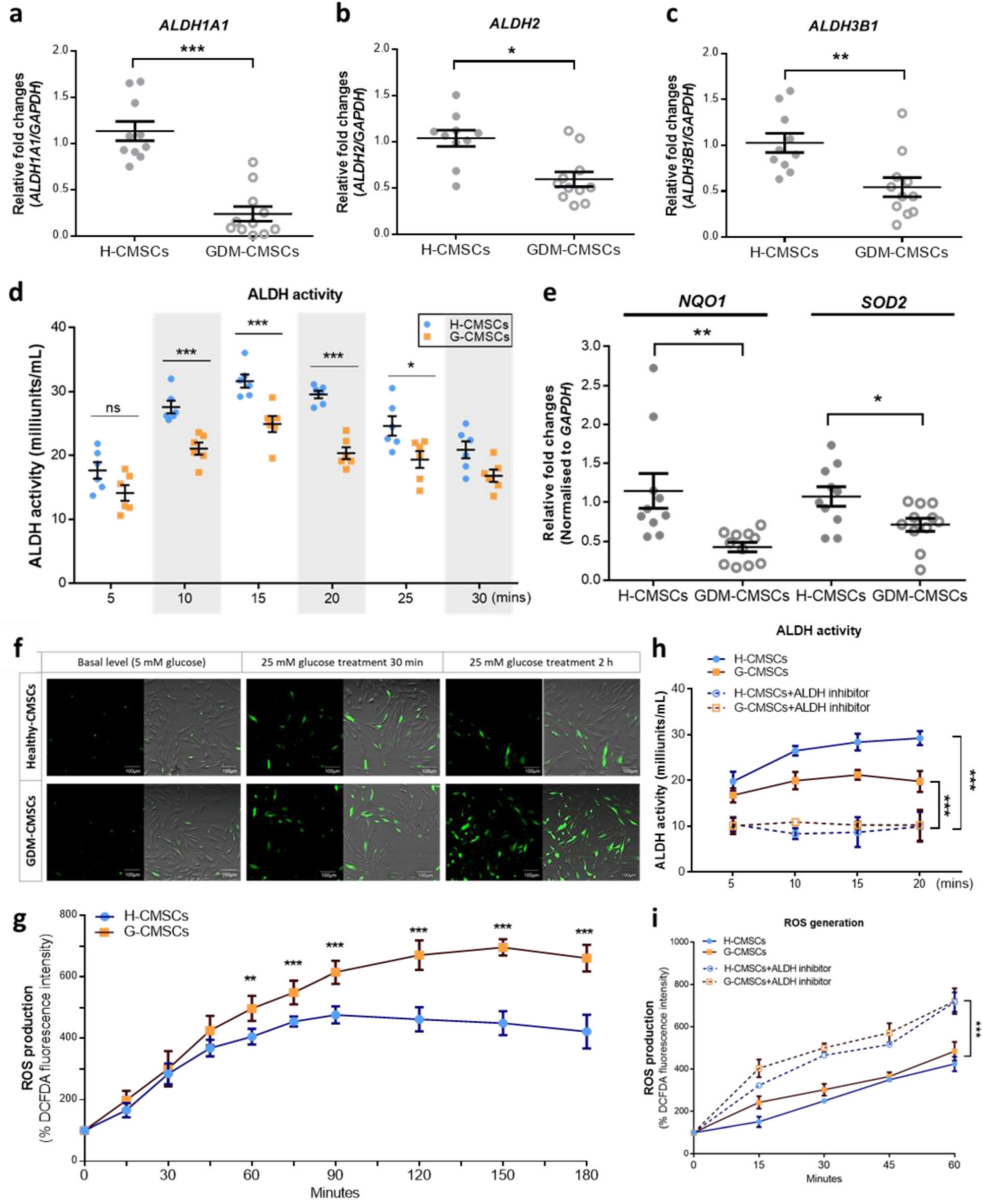
Decreased ALDH activity and increased cellular ROS level in GDM-CMSCs. **a**–**c** Expressions of *ALDH1A1*, *ALDH2*, and *ALDH3B1* were validated by real-time PCR with 10 Healthy- and 11 GDM-CMSCs samples. Gene expression levels were normalised to *GAPDH* and presented as fold change by comparing G-CMSCs to H-CMSCs using the 2^-(ΔΔCt) method. **d** ALDH activity colorimetric assay of ALDH enzymatic function in Healthy- and GDM-CMSCs. Results obtained from 6 independent experiments in duplicate. GDM-CMSCs showed reduced ALDH activity compared to Healthy-CMSCs. **e** Real-time PCR of Nrf2-mediated oxidative stress pathway genes; NQO1 and SOD2 were significantly reduced in GDM-CMSCs. Gene expression levels were normalised to *GAPDH.***f** ROS production determined by DCFDA staining (green). Representative fluorescent images of the ROS level are shown from 3 independent experiments at different time points and imaged by confocal microscopy. Scale bar 100 μm. **g** Time-course measurement of ROS production measured by fluorescent intensity. The initial fluorescence intensity at 0 min was set at 100%. Results obtained from 6 independent experiments in triplicate. **h**, **i** H-CMSCs and GDM-CMSCs ALDH function inhibited by 100 mM DEAB. ALDH enzymatic functions were examined and ROS generation evaluated by DCFDA fluorescent intensity. All error bars in this figure are presented as mean ± SEM. Student’s *t* test was used for statistical significance, *p < 0.05, **p < 0.01, ***p < 0.001

Given that the decreased expression of ALDH family genes affected several metabolic pathways and the negative activation of Nrf2-mediated oxidative stress regulation, GDM-CMSCs are likely to have imbalanced cellular ROS regulation as reflected in increased oxidative stress which are found in diabetic tissues [27]. The expression of *ALDH1A1*, *ALDH2*, and *ALDH3B1* in GDM-CMSCs and H-CMSCs was validated with real-time PCR and demonstrated significantly reduced expression levels in GDM-CMSCs (Fig. 4a–c). We further evaluated ALDH enzymatic function by ALDH colorimetric activity assay using acetaldehyde as a substrate. In the initial 5 min, there was no statistical difference in ALDH activity between two groups but significantly lower levels of ALDH activity in GDM-CMSCs were observed at 10, 15, 20, and 25 min vs. H-CMSCs. The ALDH activity gradually declined after 25 min in both H-CMSCs and GDM-CMSCs. Of note, the highest level of ALDH activity was seen at 15 min post-stimulation, in H-CMSCs (Fig. 4d).

With impaired ALDH activity, the accumulation of highly reactive and toxic aldehyde tends to induce ROS formation and increase oxidative stress [28]. Antioxidant defence reduces the harmful effects of ROS; however, a significant reduction of antioxidative enzymes, including *NQO1* and *SOD2* in GDM-CMSCs (Fig. 4e), suggested a deficiency in their antioxidant systems. To examine ROS production, H-CMSCs and GDM-CMSCs were stimulated with glucose to induce metabolic activity. Following the stimulation, ROS was produced in both H-CMSCs and GDM-CMSCs while GDM-CMSCs were observed to have higher levels of cellular ROS than H-CMSCs after 2-h glucose treatment (Fig. 4f). Measuring ROS production over a period of time, the ROS levels increased, at comparable levels, for the first 45 min in both H-CMSCs and GDM-CMSCs following stimulating with glucose. Between 45 and 90 min, ROS levels showed significantly higher rates of increase in GDM-CMSCs than in H-CMSCs. Notably, H-CMSCs did not produce increased amounts of ROS after 90 min and showed slightly reduced ROS generation whereas ROS levels in GDM-CMSCs continued to increase and remained elevated (Fig. 4g).

To further elucidate the influence of ALDH activity on ROS regulation, the enzymatic function of ALDHs was suppressed by *N*,*N*-diethylaminobenzaldehyde (DEAB), a commonly used selective inhibitor of ALDHs. In the presence of the ALDH inhibitor, the ALDH activity was significantly suppressed in both H-CMSCs and GDM-CMSCs (Fig. 4h). Moreover, when the ALDH function was suppressed, the ROS production was significantly increased during glucose-induced metabolic process (Fig. 4i). DEAB pre-treated H-/GDM-CMSCs produced higher levels of ROS than un-treated H-/GDM-CMSCs. The finding confirmed the strong association between the ALDH function and cellular ROS regulation.

Low levels of ROS are detectable in many metabolic processes; however, when ROS production is in excess of the cellular antioxidant capacity, it contributes to cellular damage. We found that downregulation of Nrf2-mediated oxidative stress regulation pathway and the impaired function of detoxifying enzymes ALDHs resulted in an insufficient capacity to respond to increased oxidative stress in GDM-CMSCs.

## Discussion

DNA microarray data provides an understanding of gene profiles and biological functions altered in GDM-CMSCs offering a valuable resource to regenerative medicine development. To conclude from our findings, the uterine environment during pregnancy could impact on the biology of stem cells derived from perinatal tissues. GDM-CMSCs, compared to H-CMSCs, displayed an enhanced migration ability and a transcriptional profile indicating potential in epithelial development and skin formation and a putative role in wound repair. In contrast, ALDH detoxification enzymes were significantly reduced in GDM-CSMCs leading to downregulation of several degradation pathways. Decreased ALDH activity in GDM-CMSCs was also associated with an impaired ability to respond to oxidative stress. Taken together, these novel findings derived from microarray and associated functional assays can be useful for exploring suitable clinical uses of CMSCs from GDM and healthy pregnancies.

GDM-CMSCs had a number of upregulated genes involved in cell motility, cytoskeleton organisation, survival, epithelial development, and skin formation. The ability of transplanted cells to mobilise and migrate to injury sites would enable a direct role in tissue repair and regeneration [29]. A recent study suggested that high glucose treatment increased human umbilical cord-derived MSC motility and promoted the migration of transplanted MSCs into mouse model wound site [30]. Human umbilical endothelial cells isolated from GDM pregnancies have also shown an increased migration ability potentially reflecting a proangiogenic GDM state [31]. In contrast, reduced wound closure capacity of perivascular stem cells from GDM women [15] and impaired migration of GDM umbilical cord-derived endothelial cells have also been reported [32]. Cell-specific impacts of GDM on motility cannot be ruled out at this time. Here we have demonstrated that GDM-CMSCs display upregulated expression of *AQP1*, *FLNB*, *CELSR1*, and *CD24*, which have important roles in cell movement and cytoskeletal remodelling, and that the GDM-MSCs have enhanced motility.

Dehydrated placental membrane has been used as a skin substitute for burned and ulcerated surfaces for many years [33, 34] while the presence of MSCs in cryopreserved placental membranes is further described as improving wound-healing therapies [35, 36]. We identified the upregulation of epithelial development-associated genes and regulators (*EDN1*, *KRT14*, *HBEGF*, *TGFB2*) in GDM-CMSCs, which may be beneficial for the clinical use as skin substitutes. The downregulation of vasculogenic factors (*RASIP1*, *CXCL12*, *RSPO3*) and the decreased activation of angiogenesis inducing pathways (IL-6, IL-8) were observed in GDM-CMSCs. It is noteworthy that hypervascularisation is frequently observed in GDM placentas due to hyperinsulinemia-mediated accelerated foetal metabolism and oxygen uptake, which leads to an imbalance in oxygen supply and demand resulting in hypoxia-induced angiogenesis [37]. Although it would be sensible to assume the involvement of GDM-CMSCs in hypervascularisation, CMSCs derived from GDM placenta did not show an increase in vasculogenesis-associated gene expression or angiogenesis pathway activity, suggesting that CMSCs may not contribute to placental hypervascularisation in GDM. On the contrary, immunohistochemistry staining of increased VEGF protein in cytotrophoblast and syncytiotrophoblast cells of GDM chronic villi [38, 39], flow cytometry identifying elevated levels of VEGF receptors on endothelial progenitor cells from GDM women [40], and increased cytokine production from Hofbauer and placental cells [41] were all associated with enhanced angiogenesis and vasculogenesis in GDM. Various cell types in GDM placenta contribute to hypervascularisation while reduced angiogenic ability in GDM-CMSCs might represent a compensatory response to counteract dysregulated angiogenesis and vascularisation or be implicated in vascular function defects of GDM placenta. In line with a recent study, a decreased in vitro tube formation ability accompanied with bFGF and VEGF downregulation was observed in GDM chorionic villus-derived MSCs [16]. Despite the high degree of angiogenesis under the hyperglycaemic environment, MSCs from GDM placenta displayed a weak angiogenic potential. This finding also pointed out the differential effects of GDM on different placental cell types during pregnancy.

Pathways associated with several degradation processes were altered in GDM-CMSCs largely due to the significant reduction of ALDHs expression. As a critical detoxification enzyme, ALDHs are highly expressed in multiple embryonic tissues, stem cells, and progenitor cells [42, 43] and protect cells against oxidative damage through detoxifying exogenous and endogenous aldehyde [28]. ALDH activity has also been used as an indicator for purification of a proangiogenic BM-MSC subset with enhanced secretory functions for vascular regeneration [44]. Significantly reduced ALDH family genes in GDM-CMSCs emphasised the adverse impacts of the pregnancy complication on cellular detoxification. GDM-CMSCs showed significantly lower ALDH enzymatic activity than H-CMSCs, which provided insufficient capacity to manage increased oxidative stress caused by hyperglycaemia. Upon stimulation, ROS production was elevated to a greater extent in GDM-CMSCs than in H-CMSCs with no sign of reduction, indicating the impaired cellular ROS regulation in GDM-CMSCs. The vicious circle of increased oxidative stress and decreased antioxidant defence progresses with gestation in GDM pathology [45, 46]. A recent study showed that treating decidual MSCs derived from preeclampsia women with an *ALDH1A1* activator restored *ALDH1A1* activity and improved H_2_O_2_-induced oxidative stress resistance in preeclampsia-derived MSCs [47]. The physiologically harmful environment of oxidative stress in GDM may be improved by antioxidant intake [48, 49]; however, restoring ALDH function itself would be a viable therapeutic target of GDM.

Developing therapeutic strategies using autologous MSCs requires a thorough understanding of their biological characteristics in vitro and in vivo. Although GDM animal models present multiple challenges due to their temporary disease condition and complex inducing factors, several studies have investigated biological properties of MSCs under type I and II diabetes mouse models. For instance, bone marrow MSCs derived from non-obese diabetic mice were found to produce high levels of proinflammatory cytokines, including IL-1α/β and MCP-1 [50]; streptozotocin-induced diabetic mice showed a reduced number of MSCs and increased apoptosis tendency [51]; and one study reported altered tri-lineage differentiation ability in bone marrow- and adipose-derived MSCs from high-fat diet-induced obese mice [52]. Various effects of diabetes on MSCs were reported over the years but the lack of comprehensive understanding impedes the development of autologous cell therapy. Moreover, relatively little is known about placenta-derived MSCs from GDM pregnancy. Our study provides a thorough analysis of genetic profiles of CMSCs from GDM and healthy women, and ultimately, in vitro functionality will require in vivo evaluation and large participation numbers to further elucidate therapeutic potential.

## Conclusion

In summary, the progression of regenerative medicine and advanced cell banking technology provides an option for the use of perinatal tissue-derived autologous MSCs for personalised medicine. The effect of GDM on CMSC gene expression was evidenced by DNA microarray analysis coupled to functional assay identification of associated impaired and enhanced functions in GDM-MSCs. These data will assist in the identification and development of suitable applications of GDM-CMSCs and eventually transform the knowledge into clinical practice.

## Supplementary information

**Additional file 1: Figure S1.** Enriched downstream cellular functions in “Physiological System Development and Function” in GDM-CMSCs.

**Additional file 2: Figure S2.** Validation of gene expression by real-time PCR.

**Additional file 3: Figure S3.** Wound healing assay.

## Data Availability

All data generated and analysed during this study are available from the corresponding author.

## Abbreviations

ALDH: Aldehyde dehydrogenase
BMP: Bone morphogenetic proteins
CD: Cluster of differentiation
CMSC: Chorionic membrane-derived stem cell
DCFDA: 2′,7′-Dichlorofluorescin diacetate
DEG: Differentially expressed gene
FGF: Fibroblast growth factor
GDM: Gestational diabetes mellitus
IL: Interleukin
IPA: Ingenuity Pathway Analysis
MSC: Mesenchymal stem cell
PCR: Polymerase chain reaction
ROS: Reactive oxygen species
STAT: Signal transducer and activator of transcription
