# Supplementary material for: Transcriptional profiling reveals altered biological characteristics of chorionic stem cells from women with gestational diabetes
Source: Stem Cell Res Ther. 2020 Jul 25;11:319. doi: 10.1186/s13287-020-01828-y (PMC7382800; doi:10.1186/s13287-020-01828-y)

Additional file 1

Figure S1. Enriched downstream cellular functions in "Physiological System Development and Function" in GDM-CMSCs

The relevant cellular processes related to development were identified and ranked by the IPA analysis based on DEGs. Activation state was calculated by IPA activation z-score.

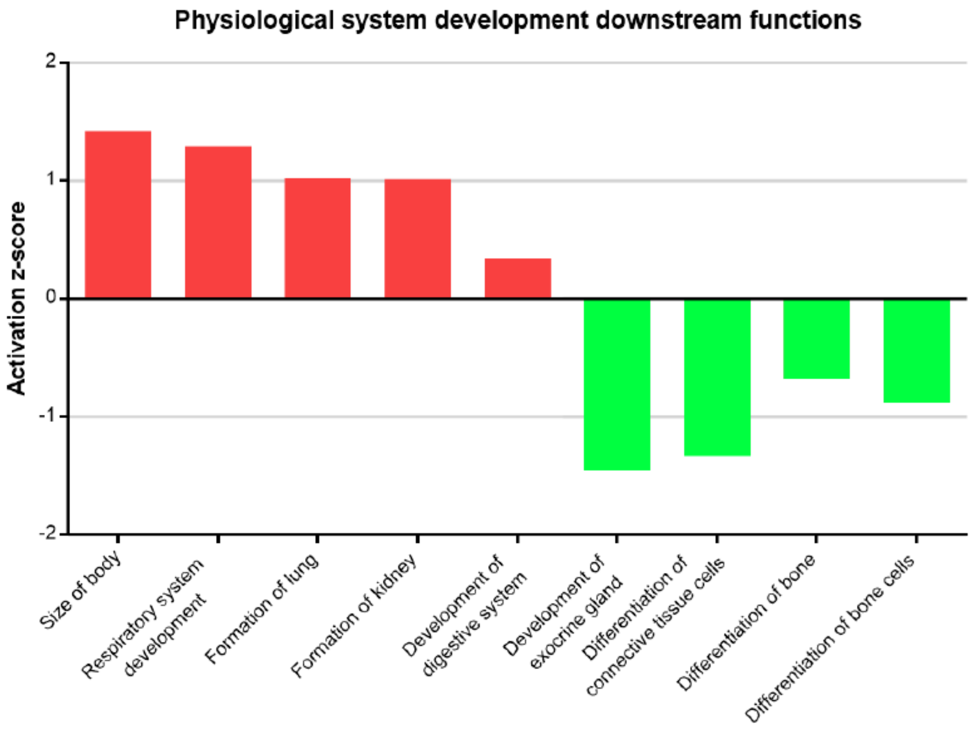

Supplement: Supplementary file 1 — Additional file 1: Figure S1. Enriched downstream cellular functions in “Physiological System Development and Function” in GDM-CMSCs. [file 13287_2020_1828_MOESM1_ESM.pdf]
