# Supplementary material for: Transcriptional profiling reveals altered biological characteristics of chorionic stem cells from women with gestational diabetes
Source: Stem Cell Res Ther. 2020 Jul 25;11:319. doi: 10.1186/s13287-020-01828-y (PMC7382800; doi:10.1186/s13287-020-01828-y)

Additional file 2

Figure S2. Validation of gene expression by real-time PCR

(a) The expression of genes involved in cell migration and wound healing – *CELSR1, TGFB2* and *CTGF* were examined by real-time PCR. (b) Cardiogenesis associated genes (*NPPB, MET*) were up-regulated while vasculogenesis associated (*HMOX1*) was significantly down-regulated in GDM-CMSCs. Expression level of each gene was normalised to *GAPDH* expression. Data are presented as mean ± SEM.

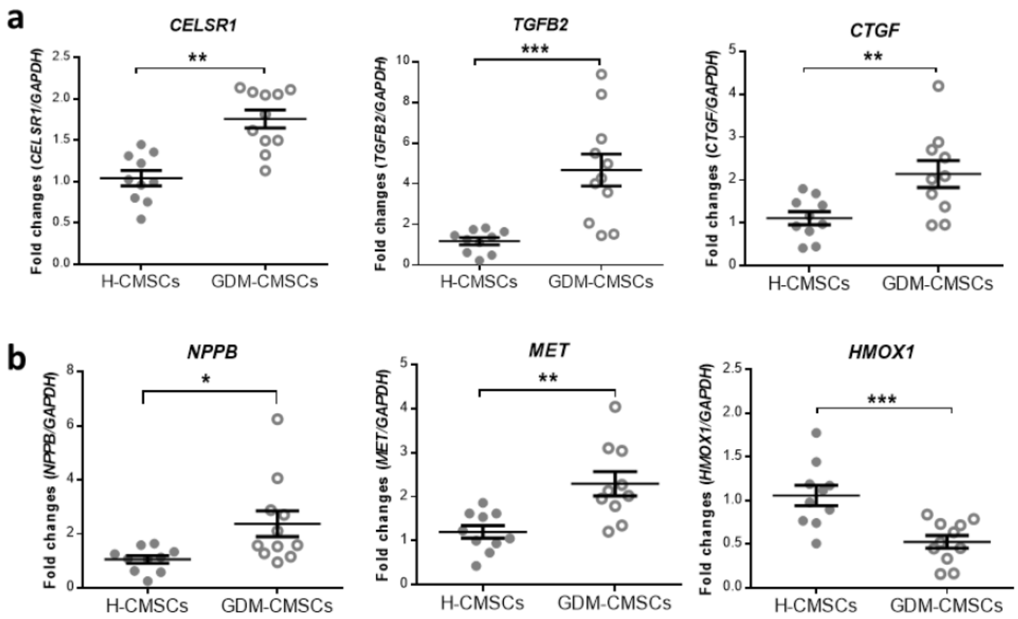

Supplement: Supplementary file 2 — Additional file 2: Figure S2. Validation of gene expression by real-time PCR. [file 13287_2020_1828_MOESM2_ESM.pdf]
