# Supplementary material for: Transcriptional profiling reveals altered biological characteristics of chorionic stem cells from women with gestational diabetes
Source: Stem Cell Res Ther. 2020 Jul 25;11:319. doi: 10.1186/s13287-020-01828-y (PMC7382800; doi:10.1186/s13287-020-01828-y)

Additional file 3

Figure S3. Wound healing assay

Representative images of wound healing assay at different time points. Numbers represent the wound area measured by Image J software. Scale bar: 150 µm. Percentage of wound closure was calculated by measuring the reduction in wound area after incubated for indicated time period. Data are presented as mean ± SEM. Statistical significance was determined by Student's t-test, $^{*}P < 0.05$, $^{**}P < 0.01$, $^{***}P < 0.001$.

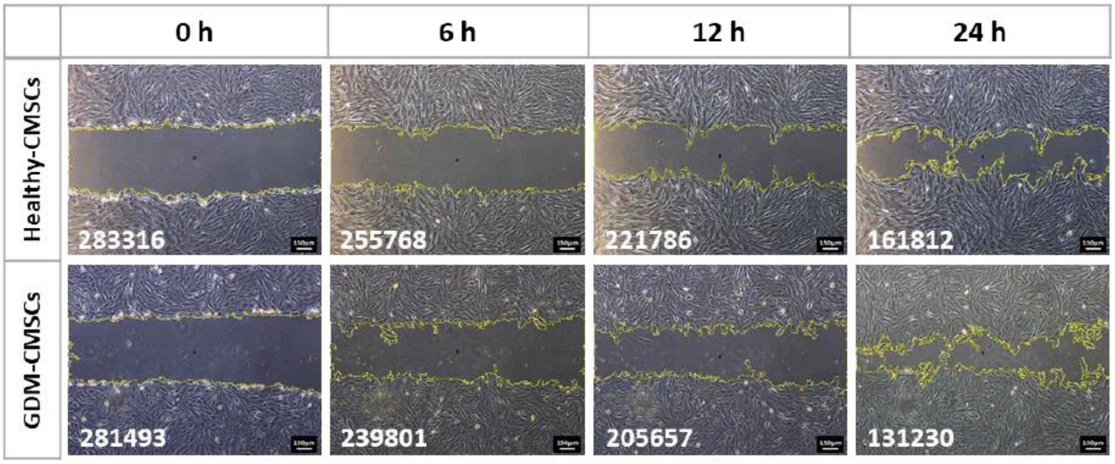

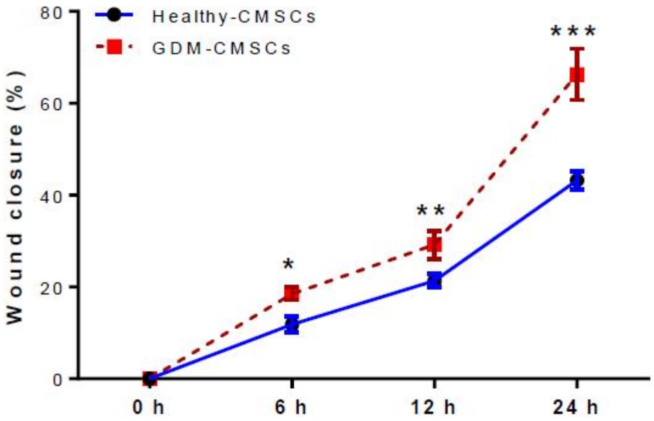

Supplement: Supplementary file 3 — Additional file 3: Figure S3. Wound healing assay. [file 13287_2020_1828_MOESM3_ESM.pdf]
